# The effects of kinesiology taping on joint stability during descending stairs in patients with acute ankle injuries

**DOI:** 10.1186/s13102-025-01321-0

**Published:** 2025-09-25

**Authors:** Ye Wei, Datao Xu, Zhifeng Zhou, Xiuye Qu, Julien S. Baker, Liangliang Xiang, Yaodong Gu

**Affiliations:** 1 Faculty of Sports Science, Ningbo University, No. 818 Fenghua Road, Jiangbei District, Ningbo, 315211 Zhejiang China; 2 KTH MoveAbility Lab, Department of Engineering Mechanics, KTH Royal Institute of Technology, Osquars backe 18, Stockholm, 10044 Sweden

**Keywords:** Acute ankle injuries, Kinesiology taping, Center of mass, Joint stiffness, The talocrural and subtalar joint displacement

## Abstract

**Background:**

Acute ankle injuries are common in sports and daily activities. Kinesiology taping enhances lower limb motion patterns, joint stability, and balance during descending stair activity in such patients. The objective of this study was to conduct a comparative analysis of the biomechanical impacts exerted by a KT group and an ST group on the lower limbs of patients with acute ankle injuries while descending stairs.

**Methods:**

The study included 27 participants with acute ankle injuries, who underwent biomechanical assessment under both KT and ST conditions. An integrated Vicon motion capture system, AMTI force platform, and electromyography (EMG) sensors were utilized to comprehensively evaluate biomechanical performance. Participants completed 20 descending stair trials under each condition, with joint stiffness, center of mass (COM), and bone displacement identified as key metrics for assessing stability. Statistical analyses, including paired t-tests and statistical parametric mapping (SPM1D), were employed to identify significant biomechanical differences between the two conditions.

**Results:**

The study revealed that compared to ST, kinesiology taping significantly reduced inversion and eversion angles (*p* < 0.001). Electromyography (EMG) analysis of the KT group revealed a remarkable enhancement in the activation levels of the calf muscle group (*p* < 0.001). In stark contrast to the ST group, the KT group demonstrated a substantial increase in ankle joint stiffness. Moreover, the KT group also achieved elevation in the activation of the hip muscles, with all these differences being highly significant (*p* < 0.001).

**Conclusion:**

Research shows that individuals with acute ankle joint injuries face issues such as significant inversion and eversion angles, reduced ankle joint stiffness, and persistent joint instability when descending stairs. Kinesiology taping can address these issues by reducing joint angles, increasing stiffness, and balancing moments. KT also stabilizes the center of mass and diminishes fall risks. This demonstrates that kinesiology taping effectively enhances stability during descending stairs and helps prevent recurrent injures. It is recommended as a protective measure following acute ankle injuries.

**Trial registration:**

ClinicalTrials.gov NCT06936033, registered on April 19, 2025 (Retrospectively registered).

**Supplementary Information:**

The online version contains supplementary material available at 10.1186/s13102-025-01321-0.

## Background

Acute ankle injuries (ATIs) are among the most common injuries experienced by athletes, particularly in sports like football, basketball, and running. Research indicates that acute ankle injuries, particularly those sustained during high-intensity sports, are associated with an increased risk of long-term functional instability in athletes [1]. Maresh reviewed the functional instability that may result after ankle ligament injury and emphasized the importance of early intervention and effective rehabilitation of acute ankle injuries [2]. Biomechanical studies have further revealed the impact of acute ankle injuries on the kinetics stability of the lower limbs [1]. Acute ankle injuries may affect the motion patterns of the ankle joint and its adjacent joints (knee, hip), resulting in decreased stability during motion and increased risk of other injuries. In addition, Wang, et al. studied the biomechanics of the knee joint through step width and proposed the idea of joints working together during motion, which is also of great significance for the understanding of acute ankle joint injuries [3]. To minimize the risk of acute ankle injuries, Willson conducted a systematic review highlighting several prevention strategies. These include the use of ankle braces by athletes, careful selection of appropriate sports footwear, and the optimization of training programs. Together, these measures aim to effectively prevent and reduce the incidence of such injuries [4]. Therefore, it is important to explore theoretical and practical significance to explore how to improve the stability of the ankle joint through effective means, reduce the risk of sports injuries, and improve body stability.

Among the many experimental research publications that explore the kinematic characteristics, kinetics mechanisms, and joint stiffness changes in patients with acute ankle injuries during climbing and descending stairs, multiple studies have explored the impact of different factors on ankle joint stability [5]. Research has revealed that there is a close relationship between changes in ankle flexion and extension angles and gait efficiency and stability [6]. Wang et al. focused their research perspective on the impact of step width on the biomechanical properties of the knee joint and emphasized that the synergy between the ankle joint and the knee joint plays an indispensable role in maintaining overall stability [3]. Protopapadaki et al. conducted a comprehensive analysis of the changes in flexion-extension angles and moments of the ankle joint during ascending and descending stair walking. They highlighted that a greater ankle flexion angle during descending stair actions may significantly elevate the risk of ankle joint injury [7]. The study by Riener et al. focused on the maximum flexion angle of the knee joint when descending stairs and found that there is a significant correlation between the angle change of the knee joint, the range of motion, and the load borne by the ankle joint [8]. Novak and Brouwer analyzed the fluctuations of joint moments during the process of ascending and descending stairs, revealing that the increase in ankle joint load in the elderly may induce the risk of injury [9]. The study by Gill et al. further explored the intrinsic connection between trunk tension and postural balance, emphasizing the important role of coordination between the trunk and lower limbs in maintaining ankle joint stability [10]. Major et al. examined the impact of prosthetic foot stiffness on gait and muscle function and pointed out that similar stiffness changes may also have a profound impact on the ankle joint stability of healthy individuals [11].

Kinesiology Taping (K-Taping), as a technology commonly used in sports medicine and rehabilitation treatment and has been widely used around the world in recent years. Through its unique elastic material design and fitting technique, k-taping provides additional support and protection for muscles and joints without restricting joint motion, thereby enhancing muscle contraction, improving endurance, reducing tremors and pain, and ultimately improving joint stability [12]. However, although k-taping is increasingly used in the field of sports medicine, its specific impact on the stability and motion efficiency of specific joints (such as ankle joints) still requires further in-depth analysis. Although some studies have reported beneficial effects of KT on joint range of motion, muscle strength, and dynamic balance, others have demonstrated limited or inconsistent outcomes [13]. Systematic reviews and meta-analyses offer a more nuanced perspective: Lim and Tay highlighted moderate-quality evidence suggesting that KT may lead to short-term improvements in certain performance metrics, particularly postural control and proprioceptive function [14]. In contrast, Parreira et al. concluded that KT provides minimal clinically meaningful benefits for general musculoskeletal conditions and advised caution in its interpretation [15]. These conflicting findings underscore the need for further task-specific and population-specific biomechanical investigations to clarify KT’s efficacy and mechanisms of action.

Research by Biz et al. revealed the positive impact of k-taping on the range of motion of the ankle joints during running activities, helping to improve joint stability and overall sports performance [16]. The study by Wikstrom et al. emphasized the significant role of k-taping in adjusting muscle activation patterns and joint moments in patients with acute ankle injuries, especially in controlling ankle eversion moments [17]. In the context of ascending and descending stairs, research by Paquette’s team demonstrates that k-taping can reduce the load on the ankle joint, optimizing the joint motion trajectory, and consequently lower the risk of injury [18]. Aytar et al. further explored the effect of k-taping on muscle force and joint stiffness and found that it can significantly improve muscle force in the injured area and increase joint stiffness, thereby enhancing the patient’s motion ability and stability [19]. Research by Ellis ‘s team revealed the unique role of k-taping in coordinating the kinematic patterns of the ankle joint and knee joint, helping to reduce the burden on the ankle joint and improve overall motion coordination [20]. Halseth et al. further verified the positive effect of k-taping in expanding the range of ankle joint motion and improving muscle activation levels, providing strong support for patient rehabilitation [21]. At the same time, research by Lee ‘s team observed that k-taping has a significant effect in improving ankle joint stability and muscle activation levels, especially in kinetics motions [22]. In addition, the research by Fayson et al. also showed that k-taping can improve the joint stiffness and muscle force output of the ankle joint, providing an effective means for patients with acute ankle injuries to improve lower limb stability and reduce the risk of reinjury [23].

The aim of this study was to explore the effects of KT and ST on the biomechanics of patients with acute injuries using a descending stairs task. We hypothesized that through the intervention of intramuscular patching, the stability of the ankle joint of k-taping patients would be strengthened, which would help to enhance the stability of the ankle joint and thereby better maintain balance during the process of walking down descending stairs.

## Methods

Each participant was tested under two taping conditions—kinesiology taping (KT) and no kinesiology taping (ST)—with the order of application randomized using a computer-generated sequence. For each condition, participants completed twenty separate descending stairs trials. To minimize potential bias, all outcome assessments, including motion capture, electromyography (EMG), and imaging analysis, were conducted by an assessor blinded to the taping condition. Participant blinding was not feasible owing to the visible and tactile differences between the two taping conditions.

A Vicon Motion Capture System, featuring eight cameras, was integrated with the AMTI Force Platform to concurrently gather biomechanical data. This data encompassed joint angles, moments, and power during the pre-weight-bearing, mid-stance, and push-off phases of the affected limb, as documented in reference [24]. Additionally, Electromyography (EMG) equipment from Delays, Boston, MA, USA, was employed to assess muscle activation patterns, as described in reference [25].

### Participants

A priori power analysis was conducted using G*Power (version 3.1.9.7; Düsseldorf, Germany) to determine the required sample size. A minimum of 27 participants was deemed necessary to detect a moderate effect size (0.50) with 80% power at $\:\alpha\:$ = 0.05 [26]. Participants were recruited through bulletin board advertisements and clinician referrals at Ningbo University. A total of 27 recreationally active college students with unilateral acute ankle sprains were enrolled (age range: 31.30 $\:\pm\:$ 12.80 years; height: 170.9 $\:\pm\:$ 8.3 cm; weight: 66.4 $\:\pm\:$ 12.2 kg). Inclusion criteria included: (1) one or more acute ankle sprains occurring within the previous 7 days, (2) ongoing symptoms of pain or instability during daily or sports activities, (3) no history of major lower limb conditions (e.g., arthritis, knee surgery), (4) AOFAS score $\:<$ 80 and VAS $\:\le\:$ 3 on the affected side, and (5) normal daily activity levels. Exclusion criteria included: (1) history of ankle surgery or significant structural damage (e.g., ligament reconstruction), (2) diagnosed neuromuscular disorders or pregnancy, (3) allergy to kinesiology tape or history of skin conditions, and (4) engagement in structured rehabilitation within the past 6 months.

All participants provided written informed consent before enrollment. The study protocol was approved by the Ethics Committee of Ningbo University (Approval No.: TY2025004), and all procedures conformed to the Declaration of Helsinki.

### Data collection procedures

All the experimental tests were conducted within the Exercise Biomechanics Laboratory of the Institute of Greater Health, situated at Ningbo University in Ningbo, China. The laboratory is outfitted with a sophisticated Vicon motion capture system (Oxford Metrics Ltd, Oxford, UK). This state-of-the-art system incorporates eight high-precision cameras, which were employed to accurately record three-dimensional motion data as the participants were engaged in the activity of descending stairs. The sampling frequency for the Vicon motion capture system (Oxford Metrics Ltd, Oxford, UK) was configured at 200 Hz [27, 28], and the system underwent meticulous calibration before each test to ensure precise measurements of joint kinematics and motion. Calibration included positioning reflective markers at predefined locations within the capture area and adjusting the cameras for optimal alignment and accuracy. This procedure was repeated at the start of each data collection session to maintain consistency in measurements.

The force platform (Watertown, Massachusetts, USA) was configured with a sampling frequency of 1000 Hz. This specific frequency setting was deliberately chosen to ensure a precise and detailed recording of ground reaction forces as the participants descended the stairs. Through this high sampling rate, any subtle variations and dynamic changes in the forces exerted on the ground during the stair - descent process could be accurately captured and analyzed. The two systems were precisely synchronized to ensure seamless data collection. The initial contact point during the descending stairs was determined based on a specific criterion. It was identified when the vertical ground reaction force registered a value exceeding 10 Nm. This standardized method of identifying the initial contact point allowed for consistent and accurate data analysis, facilitating a more in-depth understanding of the biomechanical processes involved in descending stairs. The AMTI force platform was also calibrated before each test following the manufacturer’s instructions, which involved applying a known load to verify and adjust the force output for accurate readings [29, 30].

**Fig. 1:**
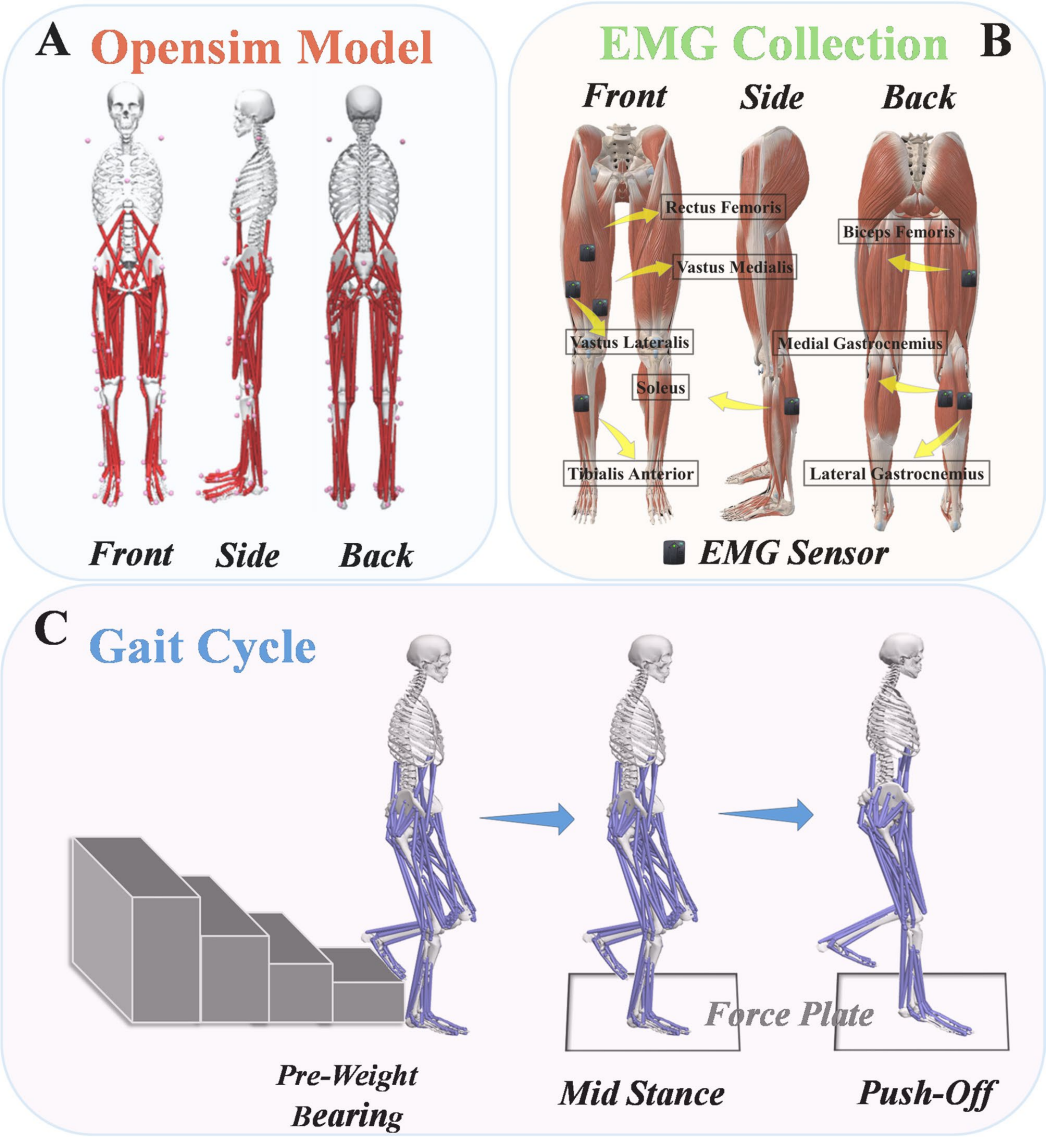
Illustrative depiction of the experimental setup and marker placement. (**A**) Anatomical locations of reflective markers on the musculoskeletal model, used for motion capture during descending stairs. (**B**) Placement of surface EMG electrodes on the major lower limb muscles of human participants. (**C**) Schematic overview of the biomechanical experiment involving descending stairs under acute injury conditions

All participants wore standardized tight-fitting clothing and remained barefoot to ensure the visibility of body markers used for motion capture. Anthropometric measurements, including height, weight, and leg length, were documented. A total of 38 markers, each 14 mm in diameter, were placed on the lower limbs and torso following the Opensim Gait-2392 model [31]. Reflective markers were placed on specific anatomical landmarks, as illustrated in Fig. 1A The placement of EMG sensors adhered to the SENIAM guidelines, with eight sensors positioned on the muscle groups of the soleus, medial and lateral gastrocnemius, tibialis anterior, rectus femoris, and medial and lateral vastus muscles, as shown in Fig. 1B The EMG system, used to capture muscle activation data, underwent calibration using standardized protocols, which included verifying sensor signal accuracy and optimizing the system to minimize noise and ensure consistent data acquisition. To enhance signal quality, skin preparation techniques such as shaving and the application of cleansing gels were performed. The EMG system’s reliability in recording muscle activity during exercise has been validated in previous studies [32].

The Vicon motion capture system, the AMTI force platform, and the EMG system were synchronized to enable integrated data acquisition. Before starting the experiment, a static calibration procedure was conducted to support the subsequent creation of manikins. Participants were first introduced to the experimental conditions and procedures. During static data collection, participants stood on the force platform with their feet al.igned parallel to the y-axis, arms extended at a 45° angle from their sides and gazed fixed forward. This posture was maintained until the static data collection was complete. The RLA gait analysis method of Rancho Los Amigos (RLA) Medical Center, California, USA was used [33]. In this paper, the gait cycle was finally divided into three phases as shown in Fig. 1C: (1) the plantar landing of the affected foot, a pre-bearing period, (2) the affected foot, a mid-stance period, and (3) the affected foot, a stomping away from the foot.

In this study, 5 cm wide KinesioTaping (Kinesio^®^Tex GOLDTM) were applied to the participants, which were characterized as being able to be stretched to 140% of their original length [34]. The K-Taping technique was applied following the methodology outlined by Jackson et al. [35]. This experiment used a randomized grouping and crossover design to divide the subjects into KT group and ST group. KT group used K-Taping (four tapes were applied to the foot (the foot was kept in dorsiflexion at 90 degrees) to provide support and stability. The first tape was anchored at the midpoint below the heel bone, with 20% tension applied at both ends to extend up the sides of the heel and talus, and slightly rotate over the tibialis anterior muscle to minimize side-to-side ankle rotation; the second tape was applied laterally at 50% tension to the lateral heel bone, extending around the heel to the medial first metatarsal bone to limit foot rotation; and the third tape, again at 50% tension, was applied to the medial talus, wrapped around the heel, and wrapped around the medial first metatarsal bone to limit foot rotation.

The third tape, also at 50% tension, starts at the medial talus, wraps around the heel and over the fourth and fifth metatarsals to further enhance plantar stability; the fourth tape is anchored from the navicular bone, wraps around the plantar foot to the lateral cuneiform bone at 20% tension, then increases to 80% tension around the Achilles tendon posteriorly to anchor it to the lateral ankle, and finally extends at 50% tension to cover the first and second metatarsal bones and wraps around the entire area. (This method of applying the patch is suitable for the protection and support needs in sports through the precise distribution of tension and directional control to fully limit the rotation and turning of the foot, and at the same time to strengthen the stability of the ankle joint and the sole of the foot) In ST group, the muscle patch was not used, and only the routine test was performed. The experimental task was a descending stairs test, which was done on a four-step staircase (each step was 300 mm in length, 900 mm in width, and 170 mm in height), and subjects were asked to complete the task of descending the staircase 20 times with a natural gait in each experimental condition. All subjects were required to be barefoot. The test was divided into two conditions, KT group and ST group, with a 5 to 10-minute break between conditions to avoid fatigue interference. Kinematic data, descending stairs time and gait stability were recorded during the experiment, and subjective perception was collected. At the end of the experiment, the muscle patch was removed, the patch site was cleaned, and the data were organized and backed up to provide a basis for subsequent analysis. The safety of the subjects was ensured throughout the experiment to avoid accidents.

To gather biomechanical data and ensure that subjects accurately complete the descending stairs task, the study will first schedule subjects to perform a standardized warm-up to ensure that the lower extremity muscles are fully activated. Prior to the start of the test, subjects familiarized themselves with the descending stairs maneuver by practicing and marking clear start and end positions on the stairs to ensure a consistent pace for each test. Data for each test was collected using a high-precision motion capture system and force platform, mainly involving changes in knee and ankle joint angles, EMG signals and joint forces. If the subject failed to complete the motion or the motion was not standardized, it was considered a failure and was not counted in the data analysis.

### High-speed biplane fluorescence fluoroscopy imaging system

**Fig. 2:**
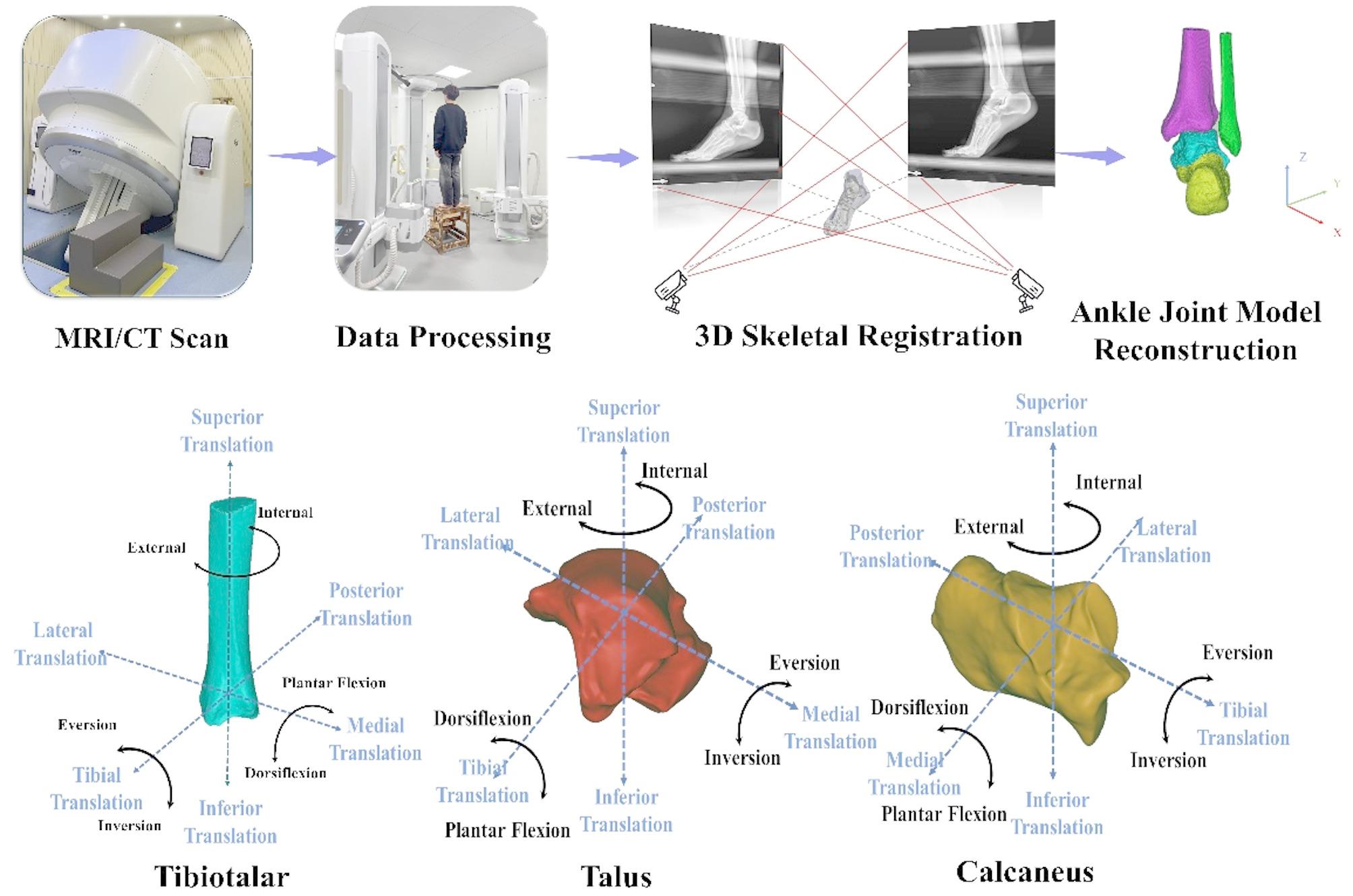
Dual Fluoroscopic Imaging System (DFIS) capturing in vivo bone motion. Shown are representative frames illustrating the motion of the tibia (left), talus (center), and calcaneus (right) during the dynamic task

High-speed biplane fluorescence fluoroscopy imaging system (DFIS) comprises a motion fluoroscopy system and a data resolution system. The former consists of two high-voltage emitters and light sources, two movable robotic arms with fluorescence receivers and intensifiers, and two high-speed cameras (Figure. 2). The distances between the 2 high-voltage emitters and receivers are 132.2 cm and 128.6 cm, respectively, with an angle of 119.6° between the image receivers; the shooting parameters were set as follows: a voltage of 60 KV, a current of 63 MA, a shooting frequency of 100 Hz, an exposure speed of 1/1000 s, and an image resolution of 1024 * 1024 pixels.

The environment calibration file generated by XMAlab was imported into Rhinoceros software for further processing. Its modeling module was used to reconstruct the shooting space and restore the relative positions of the two pairs of fluorescence emitters and image receivers in the virtual space; meanwhile, the aberration-calibrated foot and ankle X-ray images and the 3D models of the tibia, the talus, and the calcaneus were imported. The coordinate systems of the tibia, talus and heel were established according to previous standards, and the tibial-posterior, lateral-medial and superior-inferior directions corresponded to the x-axis, y-axis and z-axis, respectively, whereas plantar/dorsiflexion, inversion/eversion, and internal/external rotation were defined as the motion around the tibial-posterior, lateral-medial and superior-inferior axes, respectively [36]. The imported skeletal models were then rotated and translated in the 3D space reconstructed by the Rhinoceros software until the skeletal projections in each frame matched the skeletal contours in the fluoroscopic images.

The 6DOF data of the superior talar joint (talus versus tibia) and the inferior talar joint (heel versus talus) were calculated using the Coordinate System Calculation Plug-in Rhinoceros, which includes kinematic data in three translational (tibial-posterior, lateral-medial and superior-inferior) and three rotational (plantar/dorsiflexion, inversion/eversion, and internal/external rotation) directions (Figure. 2) [37]. Positive values represent outward, forward, and upward translation of the talus relative to the tibia (heel relative to the talus), as well as dorsiflexion, inversion, and internal rotation; negative values represent inward, backward, and downward translation, as well as plantarflexion, eversion, and external rotation.

### Data processing

Kinematic and kinetics data collected from Vicon were exported to C3D file format, then converted to a coordinate system, low pass filtered, data extracted and formatted for kinematic and ground reaction force (GRF) data using MATLAB (MathWorks, Massachusetts, USA) [38]. The C3D files were converted to TRC and MOT file formats using MATLAB and subsequently imported into OpenSim (Stanford University, Stanford, CA, USA) to compute biomechanical parameters. Musculoskeletal simulations were conducted using a model with 23 degrees of freedom and 92 muscle actuators. OpenSim’s scaling tools enabled the creation of subject-specific musculoskeletal models by adapting a generic model to align with individual body dimensions. These scaling adjustments were applied to segment lengths, segment inertia properties, and muscle attachment points. Muscle origin and insertion points, along with muscle moment arms, were tailored to match each participant’s limb dimensions. Residual moments in the frontal and lateral planes were minimized during the simulation process. The inverse kinematics tool used a weighted least squares optimization to calculate joint angles, reducing discrepancies between model-generated and experimental marker positions. The inverse kinetics tool computed joint moments for each degree of freedom, and joint power was determined by multiplying the angular velocity by the joint moment at each time step.

A static optimization algorithm was used to estimate muscle activation and muscle force, and the results were compared to surface EMG activity recorded during experiments to validate the model. The signal-to-noise ratio was optimized by performing residual analysis on a subset of data from previous studies. Kinematic and kinetic data were filtered using a fourth-order zero-lag Butterworth low-pass filter with cutoff frequencies of 12 and 20 Hz, respectively. Surface EMG signals were preprocessed by band-pass filtering with a fourth-order Butterworth filter in the frequency range of 10–400 Hz. This was followed by full-wave rectification and low-pass filtering with a cutoff frequency of 6 Hz [39]. Additionally, the EMG signals were normalized by dividing the EMG amplitude by the maximum root-mean-square (RMS) amplitude. The signals were further normalized using the maximum voluntary contraction (MVC) to determine the activation level of each muscle [40].

**Fig. 3:**
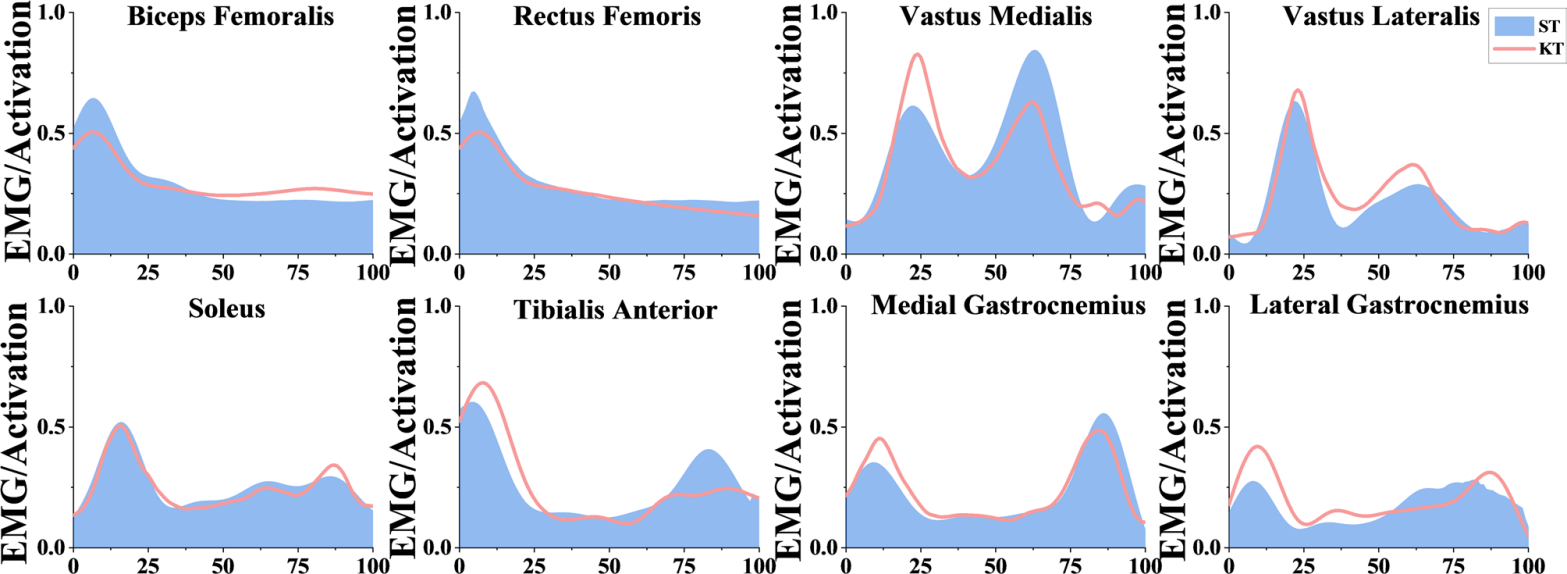
The EMG acquisition results were obtained from the recorded surface EMG signals, whereas the simulation results were derived from the musculoskeletal model implemented in OpenSim. All muscle activation levels were normalized to a scale ranging from 0 (no activation) to 1 (maximum activation), facilitating direct comparison between experimental and simulated data

The muscle activation results recorded by the EMG sensors were compared with those simulated by the musculoskeletal model to evaluate the model’s validity and accuracy. As illustrated in Fig. 3, there were no significant differences between the EMG data and the muscle activation levels predicted by the musculoskeletal model. Although originally developed for level-ground walking, the Gait2392 musculoskeletal model has been successfully applied to dynamic tasks involving greater joint flexion and loading. For example, Silder et al. employed the model to estimate muscle forces and joint kinetics during running, demonstrating its utility in high-demand locomotor activities. It remains suitable for analyzing sagittal-plane joint mechanics and lower-limb muscle forces during functional tasks such as descending stairs.

Muscle forces and activations were estimated using the Static Optimization tool in OpenSim, which minimizes the sum of squared muscle activations across all muscles at each time step, under the constraints of musculoskeletal dynamics. The optimization process used the default cost function:

1 $$\:Minimize\sum\:{a}_{i}^{2}\left(t\right)$$

Where $\:{a}_{i}\left(t\right)$ is the activation of muscle $\:\varvec{i}$ at time $\:\varvec{t}$.

The center of mass (COM) was calculated by dividing the change in moment $\:{M}_{x}$, $\:{M}_{y}$ by the change in vertical force $\:{F}_{z}$. Based on the previous study, the following equation is applied:

2 $$\:{COM}_{x}=\left(\frac{{\sum\:}_{i=1}^{n}{F}_{i,y{Z}_{i}}-{\sum\:}_{i=1}^{n}{F}_{i,z{Y}_{i}}}{{\sum\:}_{i=1}^{n}{F}_{i,z}}\right)$$

3 $${COM}_{y}=-\frac{{M}_{x}}{{F}_{z}}$$

Where $\:{M}_{x}$, $\:{M}_{y}$ denote the change of moment (about $\:x$, $\:y$ axis); $\:{F}_{z}$ denotes the change of vertical force.

Joint stiffness is determined by dividing the change in joint moment ($\:\varDelta\:\text{M}$) by the change in joint angle ($\:\varDelta\:{\uptheta\:}$). Based on previous research, the following equation was utilized:

4 $${K}_{joint}=\frac{\varDelta\:\text{M}}{\varDelta\:{\uptheta\:}}$$

Where $\:\varDelta\:\text{M}\:$denotes the change in joint moment, and $\:\varDelta\:{\uptheta\:}\:$represents the change in joint angle.

Model accuracy was enhanced using specific equations and plug-ins in OpenSim. Joint angles were calculated using an inverse kinematics algorithm, while joint moments were determined using an inverse kinetic algorithm. A residual reduction algorithm was also applied to address kinetic inconsistencies in the model. The inverse kinematics tool optimized joint angle computations by employing a weighted least squares approach to minimize discrepancies between model-predicted and experimental marker positions. Joint moments were computed for each degree of freedom in the model, and joint power was subsequently calculated as the product of angular velocity and joint moment at each time point [41].

### Statistical analysis

All statistical analyses were performed using SPSS software (version 25.0; IBM Corp., Chicago, IL, USA). To assess the normality assumption required for parametric testing, the distribution of the paired difference scores between conditions was examined using the Shapiro–Wilk test. Most of the variables demonstrated normal distributions (*p* > 0.05); thus, paired sample t-tests were applied to evaluate within-subject differences. A significance level of *p* < 0.05 was adopted for all comparisons.

Furthermore, Statistical Parametric Mapping (SPM1D) was utilized to assess time-series differences in joint kinematics, kinetics, and electromyographic (EMG) signals across the gait cycle. For variables exhibiting significant effects, Cohen’s d was calculated to quantify the effect size and interpret the practical significance of the findings.

## Results

### Joint angle, moment and power

**Fig. 4:**
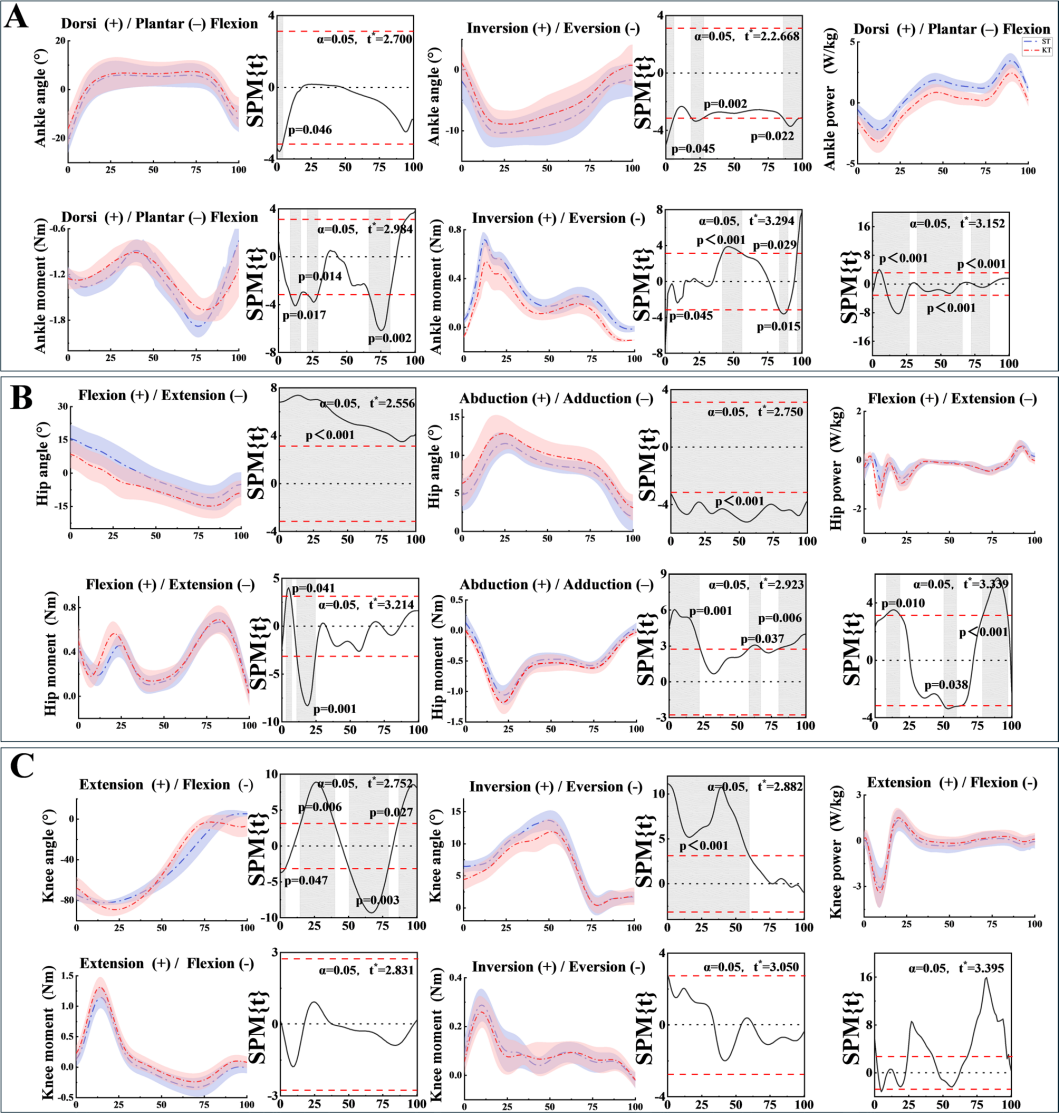
Statistical Parametric Mapping (SPM) results of lower limb joint kinematics during descending stairs. (**A**) Ankle joint results. SPM trajectories for the ST (control) and KT (experimental) groups are displayed. Blue and red lines represent the control and experimental groups, respectively. (**B**) Hip joint results under the same task condition. (**C**) Knee joint results during descending stairs

Joint angle analysis (Figure. 4) demonstrated significant differences between the KT and ST groups during descending stairs. In comparison to the ST group, the KT group displayed a notable decrease in ankle eversion angle, which was reduced from − 10.71° to -9.41°. With a consistent decreasing trend observed throughout the KT group (*p* < 0.001). In addition, the KT group demonstrated an increase in ankle eversion angles throughout the 0%-100% phase of descent (*p* < 0.001). In the KT group, the ankle dorsiflexion angle decreased significantly in the 0%-70% phase (*p* < 0.001), whereas the extension angle decreased in the 40%-100% phase (*p* < 0.001). Conversely, no significant change was detected in the dorsiflexion angle (*p* = 0.998), whereas a slight change in plantarflexion angle was detected during the 20% period. For hip angle results, KT group showed a consistent increase in hip abduction angles during descending stairs at the 0%-78% stage compared to ST group (*p* < 0.001). The KT group showed a slight increase in hip angles during descending stairs at the 0%-35% stage (*p* < 0.001), while hip flexion angle decreased in the 0%-100% range (*p* < 0.001). The analysis of knee angles during descending stairs indicated a notable decrease in knee extension angles in the KT group. This reduction was observed specifically during the 50%-100% phase of the descent. When compared to the ST group, the KT group demonstrated a significantly lower knee extension angle, with the difference reaching statistical significance (*p* < 0.001). Additionally, the KT group showed decreased abduction angles in the 0%-55% stage (*p* < 0.001). Meanwhile, no significant changes were observed in knee adduction angles (*p* = 0.954).

During descending stairs, the KT group exhibited notable increases in joint moments compared to the ST group. At the ankle joint, the dorsiflexion moment increased significantly during the 0%-90% stage (*p* < 0.001), with the plantarflexion moment rising between 23% and 29% (*p* = 0.014) and the inversion moment increasing in the 2%-21% (*p* = 0.001) and 0%-35% (*p* < 0.001) stages. The moment also exhibited a significant increase during the 42%-100% stage (*p* < 0.001). For the hip joint, the KT group showed an increase in the flexion moment during the 62%-100% stage (*p* < 0.001). Additionally, the extension moment significantly increased during the 0%-12% (*p* < 0.001) and 22%-25% (*p* = 0.012) stages. The adduction moment of the hip joint also demonstrated significant increases during the 0%-10% (*p* < 0.001), 17%-23% (*p* = 0.001), 70%-81% (*p* = 0.001), and 90%-100% (*p* < 0.001) stages. At the knee joint, the KT group showed increased flexion moments during the 0%-30% stage (*p* < 0.001), extension moments in the 50%-86% stage (*p* < 0.001), and adduction moments in the 0%-5% (*p* = 0.01), 15%-70% (*p* < 0.001), and 83%-90% (*p* = 0.003) stages.

**Fig. 5:**
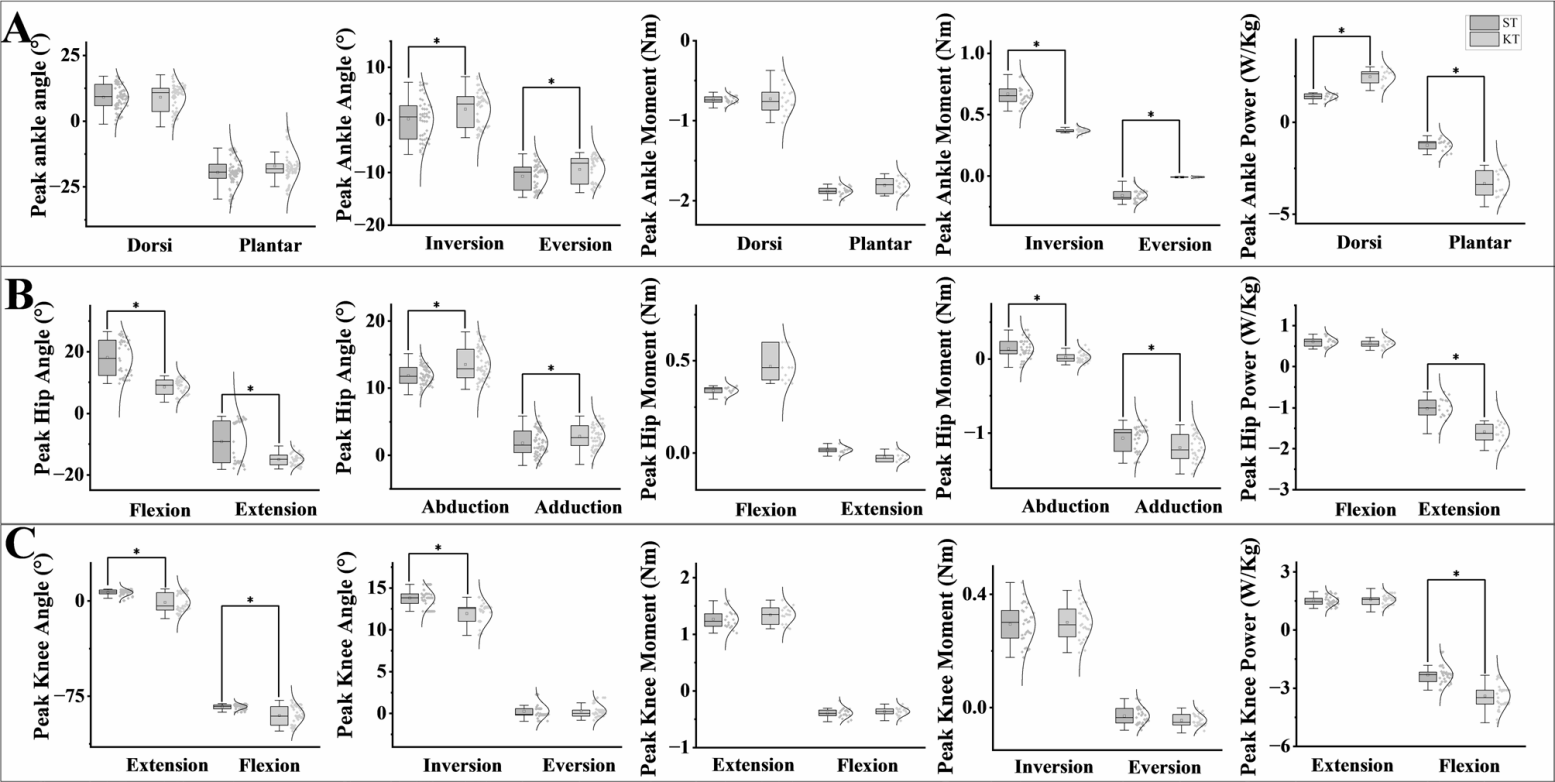
Peak values of the ankle, hip, and knee joints during descending stairs, including joint angle, joint moment, and joint power. Purple and red squares indicate the peak values of the control and experimental groups, respectively, for each joint and parameter

During descending stairs, the KT group demonstrated significant increases in joint power compared to the ST group. At the ankle joint, dorsiflexion power showed significant rises during the 0%-19% (*p* < 0.001), 33%-45% (*p* = 0.001), and 82%-94% (*p* < 0.001) phases. Similarly, plantar flexion power increased notably during the 0%-3% (*p* = 0.011) and 10%-35% (*p* < 0.001) phases. At the hip joint, flexion power significantly increased during the 0%-18% (*p* < 0.001), 40%-48% (*p* = 0.011), and 85%-95% (*p* < 0.001) phases, while extension power showed marked growth in the 20%-85% (*p* < 0.001), 32%-38% (*p* = 0.002), and 50%-85% (*p* < 0.001) phases. At the knee joint, flexion power increased during the 0%-15% (*p* < 0.001) and 67%-94% (*p* < 0.001) phases, while extension power rose significantly during the 15%-28% (*p* < 0.001) and 40%-60% (*p* < 0.001) phases. Comprehensive results on peak kinematics and kinetics are presented in Table 1; Fig. 5.

**Table 1:** Changes in ankle, hip, and knee joint angles, moments, and power in the sagittal and coronal planes before and after muscle patching. Data are presented as mean $\:\pm\:$ standard deviation (SD), along with corresponding 95% confidence intervals (CIs), *p*-values, and effect sizes (Cohen’s d). Comparisons reflect within-subject differences between pre- and post-intervention conditions across all measured joints and planes

Parameters	Peak value	Before (mean ± SD)	After (mean ± SD)	CIs	*P*-value	Effect Size
Ankle angle (°)	Dorsiflexion	9.73 ± 3.65	9.13 ± 3.00	[-2.47, 1.27]	0.998	0.06
	Plantarflexion	19.47 ± 6.63	17.04 ± 6.25	[-6.03, -1.17]	0.070	0.19
	Eversion	3.94 ± 1.18	3.34 ± 1.05	[-1.22, -0.22]	< 0.001*	0.26
	Inversion	10.71 ± 2.34	9.41 ± 2.06	[-3.57, 0.83]	< 0.001*	0.28
Ankle moment (Nm)	Dorsiflexion	7.37 ± 2.50	7.28 ± 2.39	[-1.46, 1.28]	0.835	0.04
	Plantarflexion	2.89 ± 0.16	2.81 ± 0.15	[-0.17, 0.01]	0.018	0.59
	Eversion	2.34 ± 0.17	2.74 ± 0.14	[0.31, 0.49]	< 0.001*	0.92
	Inversion	2.32 ± 0.19	2.02 ± 0.11	[0.06, 0.24]	0.001*	0.92
Ankle power (W/kg)	Dorsiflexion	2.59 ± 0.47	2.47 ± 0.43	[-0.36, 0.12]	< 0.001*	0.13
	Plantarflexion	1.35 ± 0.37	1.32 ± 0.32	[-0.22, 0.16]	< 0.001*	0.04
Hip angle (°)	Flexion	18.14 ± 5.94	17.50 ± 2.54	[-3.77, 2.49]	< 0.001*	0.07
	Extension	19.15 ± 6.95	18.97 ± 6.29	[-3.43, 3.07]	< 0.001*	0.01
	Abduction	13.87 ± 1.43	13.52 ± 1.40	[-1.14, 0.44]	< 0.001*	0.12
	Adduction	2.83 ± 1.90	2.80 ± 1.80	[-1.07, 1.01]	< 0.001*	0.01
Hip moment (Nm)	Flexion	0.97 ± 0.15	0.94 ± 0.13	[-0.11, 0.05]	0.002	0.34
	Extension	0.76 ± 0.14	0.74 ± 0.12	[-0.10, 0.06]	0.004	0.30
	Abduction	0.54 ± 0.21	0.42 ± 0.18	[-0.18, -0.06]	< 0.001*	0.47
	Adduction	1.27 ± 0.27	1.20 ± 0.23	[-0.21, 0.07]	< 0.001*	0.14
Hip power (W/kg)	Flexion	0.61 ± 0.15	0.57 ± 0.13	[-0.12, 0.04]	0.337	0.44
	Extension	1.72 ± 0.26	1.59 ± 0.20	[-0.18, 0.06]	< 0.001*	0.27
Knee Angle (°)	Flexion	6.75 ± 2.49	6.22 ± 2.27	[-19.27, -13.11]	< 0.001*	0.11
	Extension	93.33 ± 2.30	90.25 ± 2.27	[-3.42, -2.74]	< 0.001*	0.56
	Inversion	13.81 ± 1.37	11.95 ± 1.03	[-2.57, -1.15]	< 0.001*	0.61
	Eversion	1.36 ± 0.88	1.27 ± 0.76	[-0.22, 0.04]	0.954	0.05
Knee moment (Nm)	Flexion	1.37 ± 0.17	1.34 ± 0.15	[-0.14, 0.04]	0.133	0.09
	Extension	1.39 ± 0.19	1.37 ± 0.12	[1.31, 1.46]	0.385	0.15
	Inversion	2.39 ± 0.17	2.30 ± 0.16	[2.32, 2.46]	0.675	0.57
	Eversion	2.06 ± 0.13	2.04 ± 0.12	[1.98, 2.13]	0.016	0.37
Knee Power (W/kg)	Flexion	1.50 ± 0.28	1.47 ± 0.26	[1.39, 1.60]	0.236	0.06
	Extension	2.31 ± 0.49	2.02 ± 0.38	[1.98,2.63]	< 0.001*	0.76

Note: “*” indicates the difference between before and after the muscle patch for the descending stairs phase (*p* < 0.05)

### Muscle activation and muscle force

**Fig. 6:**
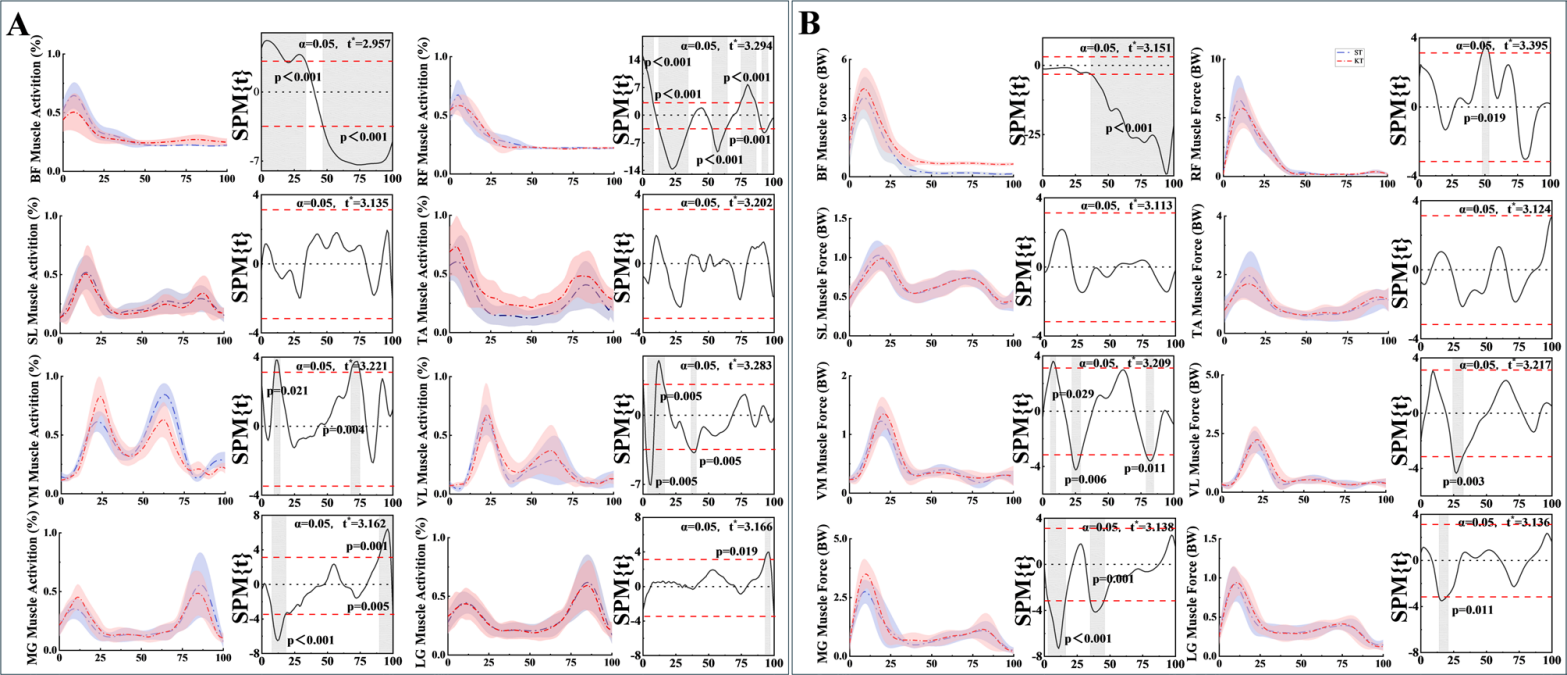
Statistical Parametric Mapping (SPM) analysis of muscle activity during descending stairs. (**A**) SPM results showing muscle activation patterns for the KT (experimental) and ST (control) groups. Blue lines represent individual muscle activation trajectories in the ST group, while red lines indicate corresponding activations in the KT group. (**B**) SPM results of muscle force profiles during descending stairs for both KT and ST groups

KT group showed differences in biceps femoris (Figure. 6) during descending stairs compared to ST group. KT group showed an increase in activation from 25% to 75% of the gait cycle (*p* < 0.001). For the soleus, activation levels were higher in KT group than in ST group during most of the gait cycle (*p* < 0.001). In the medial gastrocnemius, activation level in the middle part of the gait cycle (36%-92%) increased in KT group (*p* < 0.001), whereas in the interval 16%-28%, the activation level was lower in KT group than in ST group (*p* < 0.05). For the lateral gastrocnemius, the activation level was higher in KT group than in ST group (*p* < 0.05) at the beginning of the gait (2%-11%) but decreased in KT group (*p* < 0.001) at the end of the gait (87%-100%). Activation of the vastus lateralis increased during 24%-84% in KT group (*p* < 0.001).

The analysis of muscle force trends (BW) during descending stairs reveals distinct differences between the KT and ST groups (Figure. 6). The biceps femoris exhibited a significant increase in the KT group, particularly during 25%-75% of the gait cycle (*p* < 0.001). The rectus femoris showed smoother changes, with notable differences between the groups. For most of the gait cycle, soleus was slightly higher in the KT group, though the difference was not statistically significant. Medial gastrocnemius showed a significant increase during 36%-92% of the gait cycle (*p* < 0.001), while lateral gastrocnemius increased during 2%-11% (*p* < 0.05) but decreased at the end of the gait cycle (87%-100%, *p* < 0.001). Vastus medialis demonstrated a significant rise in force during the middle of the gait cycle (20%-80%, *p* < 0.001), and vastus lateralis increased significantly between 24% and 84% of the gait cycle (*p* < 0.001). Detailed results of peak muscle activation and force are provided in Table 2.

**Table 2:** Comparison of muscle forces pre- and post-muscle patching for each individual muscle. Values are reported as mean ± standard deviation (SD) with corresponding 95% confidence intervals (CIs). Paired comparisons were conducted to determine statistical significance (*p*-values) and quantify effect sizes using cohen’s d

Parameters Muscle force (BW)	Before (mean ± SD)	After (mean ± SD)	CIs	*P*-value	Effect size
Biceps femoris	4.19 ± 1.80	3.65 ± 1.72	[3,47, 4.90]	0.009	0.04
	4.06 ± 1.08	4.05 ± 1.07	[2.97, 4.33]	0.755	0.02
Rectus femoris	6.76 ± 2.88	6.06 ± 2.60	[5.88, 7.63]	0.124	0.13
	6.11 ± 2.05	6.10 ± 2.03	[-0.15, 0.13]	0.116	0.01
Vastus medialis	1.48 ± 0.25	1.27 ± 0.24	[1.41, 1.54]	0.001*	0.39
	1.12 ± 0.14	1.11 ± 0.13	[-0.03, 0.01]	0.34	0.04
Vastus lateralis	2.45 ± 0.49	2.19 ± 0.46	[-0.38, -0.14]	0.027	0.26
	2.18 ± 0.39	2.12 ± 0.36	[-0.10, 0.02]	0.925	0.08
Medial gastrocnemius	3.66 ± 0.62	2.91 ± 0.51	[-0.92, -0.58]	0.000*	0.55
	3.18 ± 0.52	3.12 ± 0.50	[-0.12, 0.00]	0.164	0.06
Lateral gastrocnemius	1.02 ± 0.20	0.95 ± 0.19	[-0.10, 0.04]	0.134	0.18
	1.12 ± 0.25	1.10 ± 0.24	[-0.04, 0.00]	0.008	0.04
Soleus	1.12 ± 0.18	1.08 ± 0.13	[-0.07, 0.01]	0.278	0.13
	0.36 ± 0.10	0.35 ± 0.09	[-0.02, 0.01]	0.914	0.05
Tibialis anterior	2.21 ± 0.95	2.00 ± 0.56	[-0.50, 0.08]	0.160	0.13
	0.37 ± 0.13	0.36 ± 0.12	[-0.03, 0.01]	0.543	0.04

Note: “*” indicates the difference between before and after the muscle patch for the descending stairs phase (*p* < 0.05)

### Center of mass and joint stiffness

**Fig. 7:**
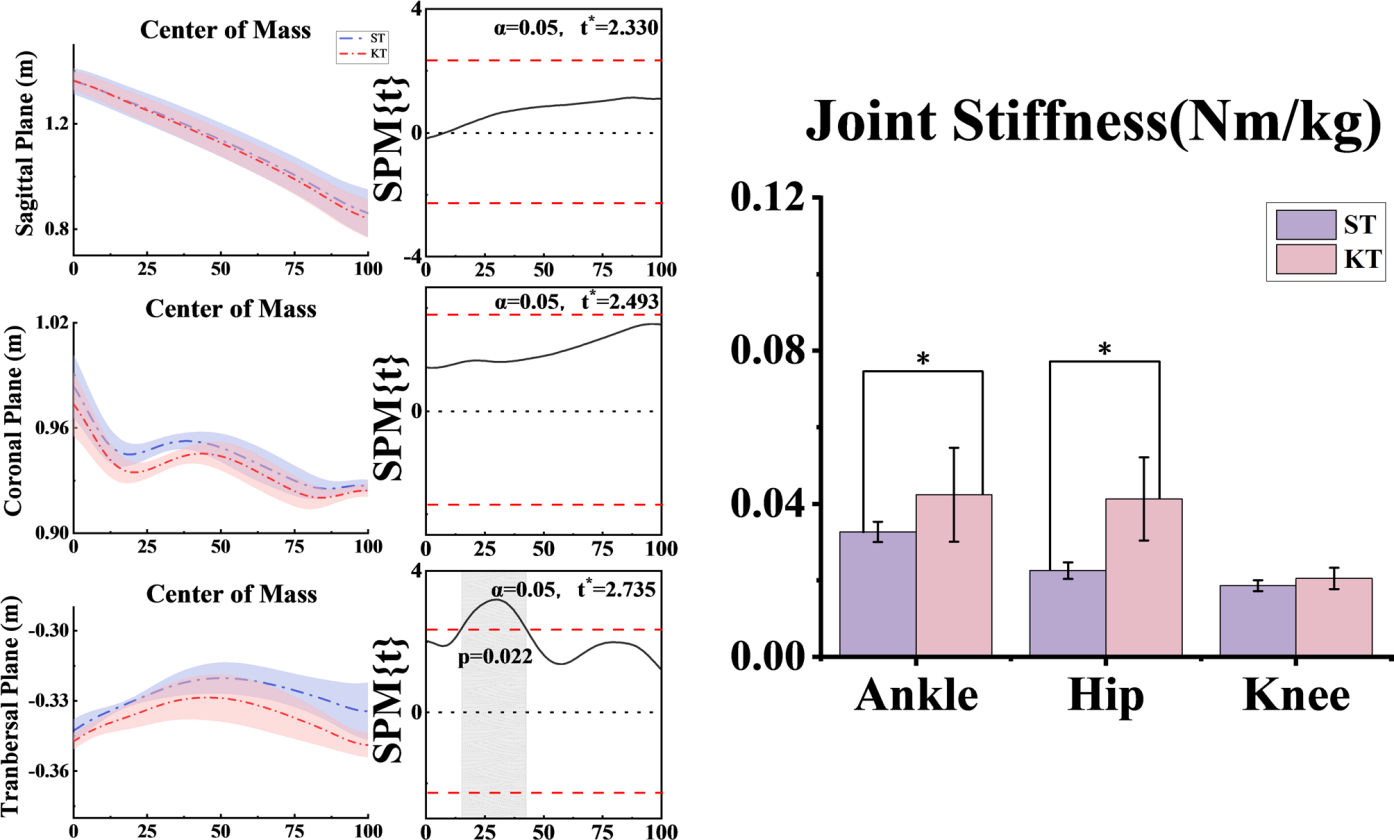
Distributions of center of mass (COM) position and joint stiffness measured during the staircase descent task. Results for the ankle, hip, and knee joints are shown, with blue and red lines indicating data from the control and experimental groups, respectively

The distribution of the center of mass (COM) position during descending stairs showed distinct characteristics in the KT group (Figure. 7). In the sagittal plane, the COM shifted forward during the 0%-20% stage (*p* = 0.018) and exhibited a more stable, centralized position from 20% to 60% (*p* < 0.001). In the coronal plane, the COM moved to the left during the 10%-30% stage (*p* = 0.002) and shifted to the right between 70% and 100% (*p* < 0.001). In the tranbersal plane, the COM remained closer to the center during the 15%-45% stage (*p* = 0.001), but larger deviations occurred in the 5%-15% (*p* = 0.003) and 85%-100% (*p* < 0.001) stages.

The distribution of joint stiffness results is illustrated in Fig. 7. Ankle joint stiffness increased in KT group at the 0%-10% (*p* = 0.005) and 60%-100% (*p* < 0.001) stages. Knee joint stiffness increased in the 20%-50% stage (*p* < 0.001). Hip stiffness, on the other hand, increased throughout the gait cycle (0%-100%) (*p* < 0.001), with the greatest increase in stiffness values especially in the 10%-40% phase (*p* = 0.002).

### The talocrural joint and subtalar joint displacement

**Fig. 8:**
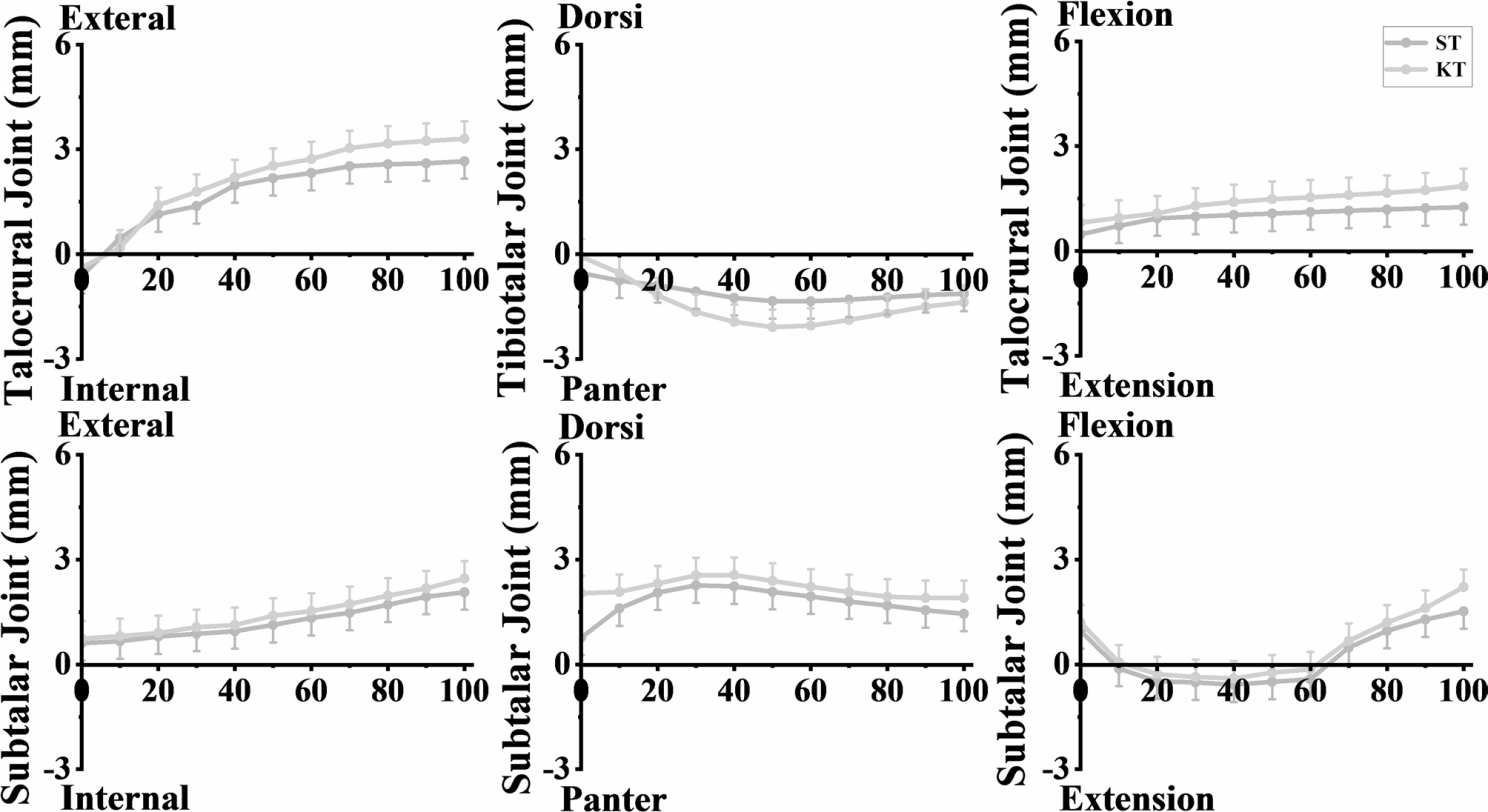
Motion Analysis of the Talocrural and Subtalar Joints

As shown in Fig. 8, the motions of KT group and ST group in the plantarflexion and dorsiflexion directions were less, and the kinematic curves exhibited variability; the motions of the talocrural joints in the flexion and extension directions were smaller; and the lines showed small changes. In the direction of plantarflexion and dorsiflexion, the motion of the subtalar joint was smaller, and the line showed little change; the internal and external rotation motions were mainly accomplished by the subtalar joint, and the line of the subtalar joint showed regular fluctuation as the motion progressed, while the activity of the talocrural joint in the direction of internal and external rotation was more obvious. Detailed results of peak the displacement of the talocrural joint and subtalar joint are shown in Table 3.

**Table 3:** The motion characteristics of the talocrural and subtalar joints before and after intervention are presented as means ± standard deviations (SD) with 95% confidence intervals (CIs). Corresponding *p*-values and effect sizes (Cohen’s d) are reported to assess significance and practical relevance

Parameters	Before (mean ± SD)	After (mean ± SD)	CIs	*P*-value	Effect size
Talocrural Sagittal Plane	3.37 ± 1.56	4.28 ± 1.35	[2.74, 4.00]	0.30	0.30
Talocrural Coronal Plane	-2.27 ± 3.52	-1.52 ± 2.57	[-5.09, 0.55]	0.12	0.12
Talocrural Tranbersal Plane	1.51 ± 1.10	0.76 ± 1.13	[-1.23, 0.27]	0.32	0.32
Subtalar Sagittal Plane	1.77 ± 1.31	2.17 ± 1.31	[-0.15, 0.95]	0.16	0.16
Subtalar Coronal Plane	1.81 ± 6.58	2.20 ± 0.22	[-1.12, 1.90]	0.04	0.04
Subtalar Tranbersal Plane	1.58 ± 2.70	2.41 ± 2.33	[-0.23, 1.89]	0.16	0.16

Note: “*” indicates the difference between before and after the muscle patch for the descending stairs phase (*p* < 0.05)

## Discussion

This study investigated the biomechanical effects of KT on individuals with acute ankle injuries during a stair descent task. The findings largely supported our initial hypothesis and revealed several key outcomes. Specifically, KT application significantly reduced ankle joint inversion and eversion angles (*p* < 0.001), indicating enhanced frontal plane control and improved ankle stability. In contrast, no significant changes were observed in dorsiflexion and plantarflexion angles (*p* > 0.05), consistent with prior findings suggesting that KT primarily influences mediolateral rather than sagittal plane motion in the ankle.

In terms of joint kinetics, KT reduced inversion torque and increased eversion moment at the ankle joints, suggesting a more favorable and stable load distribution pattern. These alterations are biomechanically meaningful, as they reduce excessive varus moments, thereby limiting ankle sprain recurrence risk [42]. Similar torque redistribution patterns have been reported at the knee, where KT application reduces internal rotation torques, enhancing proximal joint stability during dynamic tasks [43]. At the hip and knee joints, the range of motion exhibited modest reductions following KT application, likely reflecting a neuromuscular strategy shift toward greater limb control during descent [44].

These kinetic changes likely contributed to improved control of the COM, a crucial factor in dynamic balance. The KT group showed increased ankle joint stiffness, limiting excessive joint excursion and improving moment transmission efficiency. This corresponds with previous studies indicating that KT modulates joint stiffness to enhance postural stability and reduce fall risk [45]. While excessive stiffness can impair functional mobility, appropriate increases—as observed here—may help restore joint mechanics following acute injury [46].

EMG analysis further revealed that KT enhanced neuromuscular coordination, characterized by more synchronized activation across the lower limb musculature and increased activity in key stabilizers such as the biceps femoris, soleus, and both heads of the gastrocnemius. These effects support previous findings that KT enhances sensorimotor control by promoting efficient muscle recruitment and intermuscular coordination [47]. Notably, the KT group showed elevated ankle muscle activation throughout mid-stance and push-off, which may contribute to better load absorption and propulsion [48].

Additionally, KT contributed to COM stabilization during stair descent. Compared to the control group, the KT group exhibited a significant anterior COM shift in the sagittal plane (*p* = 0.018), with consistent mediolateral modulation in the coronal plane. These adaptations likely reflect changes in segmental control strategies, enabling improved balance and dynamic posture regulation. This aligns with the findings of Candela et al., who demonstrated KT’s ability to stabilize the COM during functional tasks [49].

KT also influenced lower-limb segmental interactions across the sagittal and coronal planes, promoting smoother and more controlled motion. In line with Nowak et al. [50], who highlighted KT’s role in restoring multi-planar joint motion, our results showed that the control group exhibited greater variability and asymmetry in bone displacement trajectories at the talonavicular and subtalar joints, particularly during initial contact and a stomping away from the foot. These irregularities may stem from impaired proprioception and weakened ankle musculature [51]. In contrast, the KT group demonstrated reduced kinematic variability and more congruent joint trajectories, suggesting improved sensorimotor integration and kinetic stability throughout the gait cycle.

Despite the promising findings, several limitations must be acknowledged. First, the study focused solely on immediate biomechanical responses and did not assess functional outcomes such as joint range of motion or performance-based tasks [52]. Second, the absence of longitudinal follow-up limits regarding the persistence of observed effects. Finally, although taping condition order was randomized and assessors were blinded, the visible and tactile differences between KT and standard taping could have introduced expectation-related bias.

Future studies should address these gaps by incorporating longer-term follow-ups and direct functional assessments [53]. Integration of anatomically detailed musculoskeletal models, EMG-driven simulations, and multi-task gait protocols may also yield deeper insight into KT’s mechanistic effects. Additionally, evaluating KT as part of a multimodal intervention (e.g., neuromuscular training, balance rehabilitation) may help clarify its clinical value in promoting functional recovery after ankle injuries [54].

## Conclusion

The study found that individuals with acute ankle injuries exhibit ankle joint instability during descending stairs, such as excessive inversion and eversion angles, leading to reduced balance control and increased motion trajectory fluctuations. After applying K-Taping, the inversion and eversion angles of the ankle joint were significantly reduced, and ankle stability was enhanced. K-Taping provides external support to limit excessive joint motion, improving control over inversion and eversion, and optimizes the movement trajectory of the subtalar joint. In addition, K-Taping also enables patients to have better control over bone displacement, which in turn helps to improve kinetic balance and enhance gait symmetry. This effect significantly increases stability during descending stairs and reduces the risk of recurrent sprains. Therefore, it is recommended to consider the use of K-Taping as a protective measure following acute ankle injuries. This approach not only helps restore ankle stability but also effectively optimizes lower limb motion patterns and minimizes the risk of imbalance during movement.

## Supplementary Information

Below is the link to the electronic supplementary material.

Supplementary Material 1

## Data Availability

All data generated or analysed during this study are included in this published article.

## Abbreviations

ATIs: Acute Ankle Injuries
EMG: Electromyography
COM: Center Of Mass
RLA: Rancho Los Amigos
DFIS: High-speed biplane Fluorescence Fluoroscopy Imaging System
SPM: Statistical Parametric Mapping
RMS: Root-Mean-Square
MVC: Maximum Voluntary Contraction
CIs: Confidence Intervals
